# Microglia after Seizures and in Epilepsy

**DOI:** 10.3390/cells7040026

**Published:** 2018-03-28

**Authors:** Toshimitsu Hiragi, Yuji Ikegaya, Ryuta Koyama

**Affiliations:** Laboratory of Chemical Pharmacology, Graduate School of Pharmaceutical Sciences, The University of Tokyo, Bunkyo-ku, Tokyo 113-8654, Japan; toshimitsu.hiragi@gmail.com (T.H.); yuji@ikegaya.jp (Y.I.)

**Keywords:** microglia, glia, epilepsy, epileptogenesis, seizure, synapse

## Abstract

Microglia are the resident immune cells in the brain that constitute the brain’s innate immune system. Recent studies have revealed various functions of microglia in the development and maintenance of the central nervous system (CNS) in both health and disease. However, the role of microglia in epilepsy remains largely undiscovered, partly because of the complex phenotypes of activated microglia. Activated microglia likely exert different effects on brain function depending on the phase of epileptogenesis. In this review, we mainly focus on the animal models of temporal lobe epilepsy (TLE) and discuss the proepileptic and antiepileptic roles of activated microglia in the epileptic brain. Specifically, we focus on the roles of microglia in the production of inflammatory cytokines, regulation of neurogenesis, and surveillance of the surrounding environment in epilepsy.

## 1. Introduction

It is now well recognized that microglia, as the brain’s main immune cells, play important roles in the development and maintenance of neural circuits during development and in adulthood that are unlikely to be replaceable by other cell types. Microglia actively survey the surrounding environment, regulate neurogenesis [1,2], promote survival of neurons [3], phagocytose neurons [4,5], modulate axonal wiring [6], induce synapse formation [7], and engulf unnecessary synapses [8,9]. Microglia also modify brain function through interactions with synapses [10,11] and contribute to the maintenance of synaptic function [12] and learning-dependent synapse formation [13].

**Figure 1:**
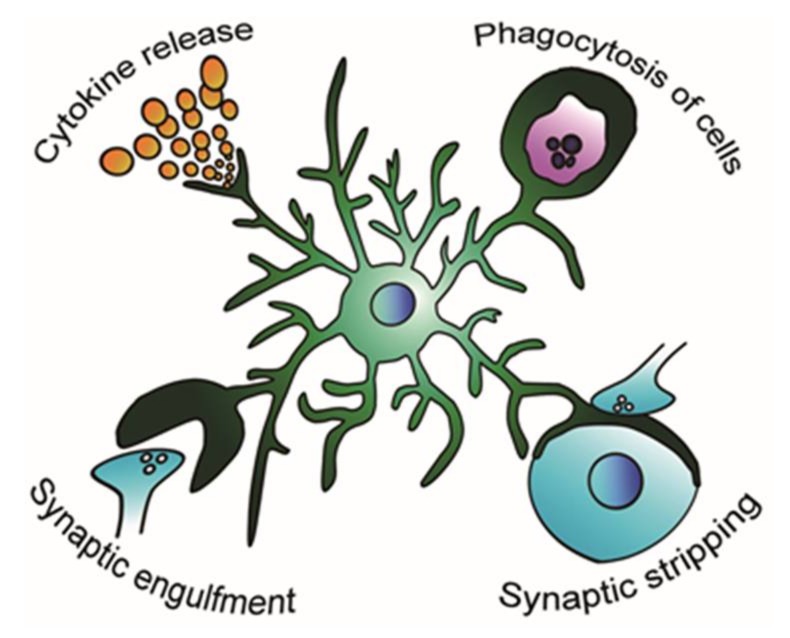
Possible modification of the microglial role in the epileptic brain. Microglia respond to the epileptic environment and change their activity of releasing proinflammatory and anti-inflammatory cytokines [16], phagocytosing apoptotic and living cells [18,19], engulfing synapses [20], and stripping synapses [21].

Despite the accumulating knowledge on microglial properties under physiological conditions, little is known about whether and how microglia modulate the structure and function of neural circuits under epileptic conditions (Figure 1). Clinical studies that have suggested anticonvulsive effects of anti-inflammatory drugs and anti-IL-1β drugs have led to the notion that inflammation contributes to epilepsy [14]. Pioneer studies support the hypothesis of an inflammatory component, including microglia, in epilepsy [15]: after acute seizures or status epilepticus (SE) induced by convulsive drugs (e.g., kainic acid (KA), pilocarpine) or electrostimulation, microglia are rapidly activated in the brain regions affected by the convulsive stimuli. Activated microglia release proinflammatory cytokines, which may lead to neuronal hyperexcitability and neurodegeneration. The microglial activation together with astrocytic activation likely contributes to the process of epileptogenesis in animal models of epilepsy, though the inflammatory state of glial cells decrease a few days after SE. It should also be noted that the microglial inflammatory state could be model dependent (e.g., KA, pilocarpine) [16]. Although a number of studies have reported that microglia are activated in patients and animal models of various types of epilepsy, the “activated” microglia have exhibited heterogeneity in their phenotypes, which makes it difficult to determine whether these microglia are proepileptic or antiepileptic. In this review, we mainly focus on temporal lobe epilepsy (TLE), which is the most common and well-studied form of epilepsy [17], and discuss the possible roles of microglia in the epileptic environment.

## 2. Activated Microglia after Seizures

### 2.1. Studies that Utilized Minocycline to Inhibit Microglial Activation

The role of “activated” microglia in the epileptic brain has been studied by using minocycline, which is considered an inhibitor of microglial activation [22]. Minocycline blocks the proliferation of microglia [23] and the expression of CD68, a member of the lysosomal/endosomal-associated membrane glycoprotein (LAMP) family. It is often used as a lysosomal marker in microglia after status epilepticus (SE) [24]. It should be noted, however, that minocycline was shown to exert neuroprotective effects on cultured neurons [25] and anti-inflammatory effects on astrocytes and oligodendrocytes [26]. Thus, the effect of minocycline can be mediated by non-microglial cells. Minocycline treatment 12 h prior to SE induced by intrahippocampal KA injection reduced the number of cleaved caspase-3^+^ apoptotic cells and TdT-mediated dUTP nick end labeling (TUNEL)^+^-damaged cells in hippocampal CA3 and CA1. This indicates that activated microglia may have a neurodegenerative role following seizures [27]. Another study showed the contribution of activated microglia to seizure severity. In a pilocarpine-induced SE model [28], consecutive 2-week treatment with minocycline from 1 day after SE reduced the number of spontaneous recurrent seizures and the duration and severity of seizures [23]. Minocycline-mediated inhibition of microglial activation also protected against developmental seizures [29]. KA-induced SE at postnatal day 25 (P25) decreased the latency to KA-induced secondary SE at P39, while minocycline treatment 1 day after the first SE restored the latency to the secondary SE. These results indicate that the first SE primed the microglia, which resulted in the reduction of seizure threshold in 2 weeks. Though the process of seizure induction is different from KA-induced SE, minocycline provoked no effect on seizure frequency or fatality rate in *Tsc1*^GFAP^CKO mice, a genetic mouse model of tuberous sclerosis complex [30]. Although it is unknown how microglia exacerbate seizures and accelerate epileptogenesis, it is possible that microglia increase neuronal activity directly or indirectly by secreting inflammatory cytokines. Because minocycline blocks the SE-induced increase in cytokine concentrations (IL-1β and TNF-α) in the hippocampus [23], it is possible that microglia initiate brain inflammation after seizures. 

It should be noted that peripheral immune cells could also contribute to neuroinflammation. In the hippocampus of TLE patients, infiltration of peripheral immune cells, including leucocytes, granulocytes, and monocytes, was evident [31,32]. The infiltration of peripheral immune cells was transiently observed after pilocarpine- or KA-induced SE in rodents [31,32]. Using the pilocarpine-induced SE model, Vinet et al. showed that microglia were less proinflammatory than infiltrating myeloid cells 24 and 96 h after SE [33]. However, in a recent study using the KA-induced SE model, similar to C-C chemokine receptor type2 (CCR2)^+^ infiltrating monocytes, microglia also expressed proinflammatory cytokines [34]. It has also been shown that a CCR2 knockout decreased the number of infiltrating monocytes, IL-1β expression in microglia, and the number of FluoroJade-B^+^ damaged neurons following SE but did not affect the severity of KA-induced acute seizures [34,35]. These results suggest that the infiltration of peripheral immune cells contribute to neuroinflammation and neurodegeneration rather than exacerbation of seizures.

### 2.2. Increases in Inflammatory Cytokines

Inflammatory cytokines, whose expression levels are often elevated in the brains of patients and animal models of TLE, are known to increase the excitability of neurons and are thus thought to be involved in epileptogenesis [14,36]. The expression of proinflammatory cytokines including IL-1β, IL-6, and TNF-α is elevated in the hippocampus within 1 day of SE induced by electric stimulation [37] and within 3 h of developmental febrile SE [38]. These inflammatory cytokines can be produced by several brain cells, including neurons, astrocytes, microglia, and endothelial cells. Recently, it was shown that activated microglia play a primary role in the production of cytokines. Benson et al. investigated microglial-specific expression of inflammatory cytokines using flow cytometry and quantitative real-time PCR after SE [16]. Microglial proinflammatory cytokines (IL-1β, IL-6, and TNF-α) showed increased expression 3 days but not 21 days after pilocarpine-induced SE. It should be noted, though, that anti-inflammatory cytokines (Arg1, IL-4 and IL-10) were also increased in microglia, indicating a complex role of microglia in the epileptic brain.

Toll-like receptor (TLR) signaling in activated microglia may mediate the production of cytokines in the epileptic brain. An in vitro study showed that microglia responded to the TLR3 agonist polyinosinic:polycytidylic acid (poly(I:C), a synthetic double-stranded RNA) and to the TLR4 agonist lipopolysaccharide (LPS), promoting the production of inflammatory cytokines [39]. TLR3 deficiency mitigated spontaneous recurrent seizures in pilocarpine-induced epileptic mice [40] and TLR4 antagonists reduced the number of acute seizures in KA-induced epileptic mice [41]. Although the endogenous ligand of TLRs in vivo has not been determined, microglia may be activated through their own TLRs and accelerate epileptogenesis. It should be noted that astrocytes have also been suggested to express TLR4 following KA-induced acute seizures [41]. Thus, it is possible that TLR4 agonist/antagonist also affects astrocytic production and release of cytokines.

A recent study using *Tsc1*^Cx3cr1^CKO mice [42], in which mTOR signaling is elevated selectively in microglia, suggested that microglia contribute to epileptogenesis without proinflammatory signals. *Tsc1*^Cx3cr1^CKO microglia exhibited reactive morphologies, increased proliferation, and upregulated lysosomal genes in vivo and increased phagocytic activity in vitro. Although the gene expression of proinflammatory cytokines was elevated in the hippocampus of *Tsc1*^Cx3cr1^CKO mice, purified microglia showed decreased expression of proinflammatory cytokines. Moreover, *Tsc1*^Cx3cr1^CKO mice developed spontaneous seizures by 5 weeks of age. Although the detailed mechanisms remain unknown, these results suggest that the upregulation of mTOR signaling in microglia may induce spontaneous seizures in a microglial proinflammatory cytokine-independent manner. It should be noted, however, that the expression of proinflammatory cytokines was examined in 4–5-week-old mice when the onset of spontaneous seizures was detected. Thus, it is possible that the proinflammatory signals in microglia were elevated long before the onset of spontaneous seizures.

Astrocytes can also modulate microglial release of cytokines. Using co-cultures of astrocytes and the murine microglial cell line N9, Bianco et al. showed that mechanical stimulations, which enhance ATP release from astrocytes, induced IL-1β release from N9 microglial cells [43]. Because ATP release is increased during seizures [44,45], the source of ATP (whether it is neuronal or astrocytic or both) being still under debate, it is possible that astrocytes could modulate microglial release of cytokines through ATP-dependent mechanisms under epileptic conditions.

### 2.3. Fractalkine Signaling

Enhanced signaling of fractalkine, i.e., chemokine (C-X3-C motif) ligand 1 (CX3CL1), also plays a role in the epileptic brain. CX3CL1 is a chemokine expressed principally by neurons and binds to the CX3CR1 receptor, which is selectively expressed on the surface of microglia [46] and mediates neuron–microglia interactions [47,48]. In TLE patients, CX3CL1 immunoreactivity and protein levels are increased in the temporal neocortex and hippocampus compared to nonepileptic autopsy controls [49]. After pilocarpine-induced SE in rats, CX3CL1 immunoreactivity was also increased in the hippocampus 1–3 h after SE and decreased 3 days after SE [50]. In contrast to the decrease in CX3CL1 immunoreactivity, the immunoreactivity of CX3CR1, the receptor of CX3CL1, remained higher than control at 3 days after SE. Neuronal damage was detected 3 days after SE but the damage was rescued by intracerebroventricular infusion of antibody against CX3CL1 or CX3CR1 (infusion was started 4 days before SE induction and continued for a week). These data suggest that the CX3CL1–CX3CR1 cascade contributes to the neurodegeneration after seizures. In contrast to the above findings, Roseti et al. reported beneficial effects of CX3CL1 in the epileptic brain. CX3CL1 treatment restored the decreased gamma amino butyric acid (GABA)-evoked currents in excitatory neurons recorded in fresh cortical slices resected from patients with temporal lobe epilepsy, which implies that CX3CL1 promotes the stability of GABA-evoked currents and thereby regulates the excitatory/inhibitory (E/I) balance of neural circuits [51]. However, no study has reported on the changes in seizure severity during SE or the development of spontaneous seizures in CX3CR1 knockout mice. Thus, the role of CX3CL1–CX3CR1 signaling in seizure severity and epileptogenesis remains to be determined.

Another chemokine receptor type 4 (CXCR4), which is expressed by microglia and astrocytes [52], may play a proepileptic role. A one-week treatment with the CXCR4 antagonist AMD3100 at 2 months after the intracerebroventricular KA injection decreased the number and duration of spontaneous electroencephalographic seizures [53]. CXCR4 is also known to mediate microglia–astrocyte interaction and modulate the function of these glial cells [54]. In the co-culture of astrocytes and microglia, stromal cell-derived factor 1 (SDF-1, also known as CXCL12), the natural ligand of CXCR4, induced microglial release of TNF-α as well as astrocytic release of glutamate. Since glutamate released from astrocytes can result in neuronal hyperexcitability [55], it is possible that CXCR4 signaling contributes to epilepsy.

## 3. Microglia and Neurogenesis after Seizures

### Microglia Modulate Aberrant Neurogenesis after Seizures

It is well recognized that SE induces aberrant neurogenesis in the subgranular zone (SGZ) of the dentate gyrus in rodents. Aberrant neurogenesis in the epileptic brain includes a significantly increased number of newly generated granule cells [56,57] and the ectopic positioning of these cells in the hilus, i.e., ectopic granule cells [58]. Newly generated granule cells in the epileptic brain, especially ectopic granule cells, can be proepileptic [59,60,61], and those incorporated in their original position, i.e., the granule cell layer, can be antiepileptic [62,63]. 

Accumulating evidence suggests that microglia regulate each step of adult neurogenesis, including proliferation, survival, and maturation of newly generated cells both in the nonepileptic and epileptic brain [64]. Ekdahl et al. induced partial or generalized SE by electric stimulation of the ventral hippocampus and examined the relationship between microglia and neurogenesis. Five weeks after partial SE, the number of both activated microglia and 4-week-old newly generated granule cells was increased. In generalized SE, although the number of activated microglia was further increased, the increase in the number of newly generated granule cells was attenuated, a phenomenon blocked by daily treatment of minocycline after SE. These results suggest that microglia differentially modulate neurogenesis according to the severity of the seizure [24]. Another study revealed that the number of microglia and the percentage of amoeboid microglia were increased 1 week after SE induced by electric stimulation of the hippocampus. Additionally, the number of DCX^+^ immature newborn neurons and Ki67^+^ proliferating cells was increased in the dentate gyrus. These SE-induced alterations were partially blocked by intracerebroventricular infusion of the antibody against CX3CR1 during the first week post-SE [65], suggesting that microglial CX3CR1 may promote the seizure-induced aberrant neurogenesis in the epileptic brain.

In contrast, a recent study found that microglia suppressed aberrant neurogenesis after seizures, as KA-induced aberrant neurogenesis was aggravated in TLR9-deficient mice [66]. In the epileptic condition, microglia used their receptor TLR9 to sense self-DNA, which was presumably released from dying neurons, and then secreted TNF-α, suppressing the proliferation of neural stem cells. Moreover, TLR9-deficient mice showed worsened behavioral seizures 48 days after the first KA injection. Thus, activated microglia may suppress aberrant neurogenesis through secretion of TNF-α, resulting in antiepileptic effects.

Microglia directly engulf apoptotic newborn cells in the dentate gyrus and maintain the homeostasis of neurogenic niches in the nonepileptic condition [2]. However, when SE was induced by intrahippocampal KA injection, the phagocytosis of cleaved caspase-3^+^ apoptotic neurons by microglia was impaired, and the balance between microglial phagocytosis and neuronal apoptosis collapsed 1 day post-SE [18]. Although microglial phagocytosis transiently recovered 3 days post-SE, the imbalance between phagocytosis and apoptosis was evident until 4 months post-SE. Abiega et al. further found that microglia purified from the hippocampus 1 day after SE showed decreased expression of receptors related to phagocytosis (TREM2, MerTK, CR3, and GPR34). In addition, it was revealed that the expression of the receptors for ATP (P2X4, P2Y6, P2Y12), which is a well-known regulator of neuron-microglia interactions [67], was increased. Because a high concentration of ATP impaired microglial phagocytosis in vitro and in vivo, Abiega et al. concluded that SE triggers neuronal hyperactivity and saturation of the ATP signal, resulting in impairment of microglia’s ability to sense degenerating neurons through ATP microgradients [18]. Another study reported a contradictory result, showing an increased phagocytic activity of microglia after SE [68]. The contradictory conclusions may result from the difference in phagocytosis assays. Abiega et al. evaluated the phagocytic activity by quantifying the percentage of engulfed apoptotic cells in the dentate gyrus [18], whereas Koizumi et al. evaluated the phagocytic activity by quantifying the number of engulfed microspheres that was injected into the hippocampal CA3 regions [68]. 

Luo et al. reported the phagocytosis of viable newborn cells in the epileptic dentate gyrus [19]. Microglia showed an activated phenotype, including increased cell density and increased expression of the lysosomal marker CD68 after KA-induced SE. These activated microglia engulfed more newly generated cells than control, resulting in the rapid removal of newly generated cells within 1 week after SE. The activated microglia engulfed not only apoptotic but also viable newly generated cells. Further, the ectopic localization of newly generated cells was increased when SE-induced microglial activation was attenuated with minocycline. 

## 4. Surveillance of the Environment by Microglia after Seizures

### 4.1. Purinergic Receptors Mediate Microglial Contacts with Neurons

Microglia move their processes around to scan the extracellular environment (basal motility) and direct their processes towards potential dangerous signals (e.g., laser lesion, pipette containing ATP analogues). SE induces morphological activation of microglia in a time-dependent manner [69]. Wyatt–Jonson et al. classified microglial morphologies into five shapes: ramified, hypertrophic, bushy, amoeboid, and rod. In the hippocampus, the bushy shape predominated 4 h after SE and the amoeboid shape predominated at 3 days and at 2 weeks after SE. Morphological changes to microglia may represent their specific function, such as phagocytosis and synaptic surveillance in the epileptic brain. Avignone et al. performed in vitro two-photon time-lapse imaging of microglial motility, revealing the dynamic changes in microglial motility after SE [70]. The application of 2-MeSADP (agonist of P2Y12 and P2Y13 receptors) to the perfusate significantly increased the motility of microglial processes in acute slices 48 h after KA-induced SE compared to non-SE control [70]. The authors further investigated the basal motility of microglial processes 48 h after KA-induced SE, finding that microglia explored a larger territory after SE, although the elongation velocity of microglial processes did not change [71]. In contrast to these findings, Abiega et al. reported a decreased basal motility of microglial processes after SE by performing both in vitro and in vivo two-photon time-lapse imaging [18]. The contradictory conclusions may result from the age of the mice, the method of seizure induction, and the timing of observation. Avignone et al. used young adult (postnatal day 30–40) mice, induced SE by KA injection (i.p.), and observed the motility of microglial processes 48 h after SE [71], whereas Abiega et al. used adult (postnatal day 60) mice, induced SE by intrahippocampal KA injection, and observed the motility of microglial processes 24 h after SE [18]. It is possible that microglial activation may regulate different types of motility in a different way; the changes in basal process motility and the directed movement of processes towards potential dangerous signals vary according to the stimulus or model [18,70,71]. A recent work from Madry et al. suggested that the basal motility and the directed motility of microglial processes could be regulated by different mechanisms. The basal motility and ramification are mediated by the two-pore domain channel THIK-1, which was shown to be the main K^+^ channel expressed in microglia, in physiological conditions, whereas the process direction towards potential dangerous signals is mediated by ATP signaling through P2Y12 receptors [72].

Purinergic receptor signaling, whose main role is related to the release of cytokines and other factors, may indirectly contribute to the dynamics of the microglial process under epileptic conditions as the number of primary processes of microglia were affected in P2Y12 KO mice [73] and the expression of microglial purinergic receptors was upregulated in the hippocampus 2 to 3 days after KA-induced SE [68,70,74]. Jimenez–Pacheco et al. showed the contribution of P2X7 receptor to epilepsy using an SE model induced by an intra-amygdala injection of KA [75,76]. The authors showed that pharmacological blocking of P2X7 receptors pre- and post-SE attenuated neuronal damage and seizure severity. It should be noted, though, that P2X7 receptors were detected in neurons and astrocytes as well as microglia [75] and that the targets of the P2X7 receptor antagonists were debatable.

Consistent with the morphological changes in microglia, neuron–microglia interactions are altered in the epileptic brain. Three days after KA-induced SE, the contact between microglia and the apical dendrites of CA1 pyramidal neurons was increased in the hippocampus [77]. Eyo et al. investigated the mechanism of recruitment of microglial processes onto neuronal elements at the scale of dendrites [73]. They showed that SE induced neuronal release of ATP, resulting in increases in microglial process motility and interactions with neuronal dendrites and soma through P2Y12 activation on microglia immediately after SE. Although the causal relation between the microglial process motility and epileptogenesis remains elusive, in P2Y12 knockout (KO) mice, the seizure score was increased and seizure threshold was decreased after KA injection, suggesting antiepileptic effects of microglial P2Y12 receptors. An in vivo imaging study using zebrafish supported the antiepileptic theory of neuron-microglia interactions [78]. This study reported that microglia preferentially contact with the cell bodies of neurons with higher spontaneous activity. Further, it was reported that microglial contacts on neurons resulted in the reduction of visually-evoked neuronal activity. Further, it has been reported that a reduction in extracellular calcium induced microglial contacts with neuronal dendrites [79] and that microglial contacts with neuronal dendrites were modulated by fractalkine signaling that was triggered by IL-1β under epileptic conditions [80]. Taken together, these findings indicate that microglia sense ATP released from hyperactive neurons through P2Y12 and interact with neuronal dendrites. Microglia could also sense neuronal CX3CL1 and release IL-1β, which in turn would increase neuronal excitability, shaping a feedback loop that enhances the neuron–microglia interaction that may help suppress the hyperactivity of neurons. 

### 4.2. Removal of Synapses by Microglia after Seizures

Microglia can directly modulate synaptic structures via synaptic engulfment and stripping in epilepsy. The classical complement pathway mediates microglia-dependent synapse elimination in both the developing and adult CNS [9,81], and the expression of complement molecules is elevated in patients with epilepsy and in animal models. In brain tissues from patients with epilepsy, increased expression of C1q [82] and its downstream product iC3b (activated form of C3) was detected [20]. Importantly, a 3D confocal microscopic analysis revealed that C1q was colocalized on neuronal dendrites, which were also colocalized with the microglial marker Iba1 [20]. In a pilocarpine-induced SE model, the expression of C1q and iC3b was increased, as was Iba1 immunoreactivity, a possible indicator of microglial activation, in the hippocampus [83]. Because the protein level of iC3b was positively correlated with the number of spontaneous seizures, it is possible that complement-dependent synaptic engulfment by microglia is associated with seizure severity. Future studies are needed to clarify whether microglial synaptic engulfment, especially the pruning of inhibitory synapses, contributes to epileptogenesis. It is also possible that stripping of inhibitory synapses by the microglial covering neuronal cell bodies, as observed in the inflamed brain [21], induces neuronal hyperactivity in epilepsy. A recent study revealed that the IL-1 family cytokine interleukin-33 (IL-33), released from astrocytes, promotes synaptic engulfment by microglia in the development of the spinal cord ventral horn [84]. The results raised the possibility that astrocytes modulate synaptic engulfment by microglia under epileptic conditions. However, the underlying mechanisms can be more complex and include several mechanisms and molecules in addition to IL-33, which should be clarified in future studies.

## 5. Conclusions

In the epileptic brain, microglia exhibit complex phenotypes that could be either proepileptic or antiepileptic. It has been suggested that short-term microglial activation is beneficial [85,86] whereas chronic microglial activation is detrimental [87] for the pathogenesis of epilepsy. It is important to determine the changing roles of microglia in each process of epileptogenesis. 

Although most studies have focused on the contribution of microglia in deteriorating seizures and epilepsy, a protective role of microglia in epileptogenesis should be also studied. It was shown that the expression of P450 cholesterol side-chain cleavage enzyme (P450scc), which catalyzes the biosynthesis of GABA_A_ receptor modulatory neurosteroids, was elevated in glial cells (especially in microglia and astrocytes) 3 days after pilocarpine-induced SE [88]. Thus, microglia may play a protective role in the early stages of epileptogenesis through enhancing GABAergic signaling. 

Given the limitations of the pharmacological approaches such as minocycline (i.e., non-specificity to microglia), both pharmacological and genetic approaches to inactivate or eliminate microglia will be necessary to further determine the selective and conditional roles of microglia after seizures and in epilepsy. Another obstacle in clarifying the role of microglia after seizures and in epilepsy is the inconsistency of the models of epilepsy, because the status of microglial activation is affected by how the epileptic seizures are induced in each model. In addition, most of the studies highlighted in this review have focused mainly on the post-SE period and not necessarily epilepsy with spontaneous seizures. This research is of importance in terms of studying the process of epileptogenesis (if SE is followed by spontaneous seizures) but research should also be directed to proper models with spontaneous seizures that are more relevant for the pathology of human epilepsy. Active future studies are awaited to clarify the role of microglia in epilepsy and the mechanisms by which microglia directly and indirectly modulate the excitatory versus inhibitory balance of synapses in the process of epileptogenesis.

## Abbreviations

CNS	central nervous system
TLE	temporal lobe epilepsy
SE	status epilepticus
KA	kainic acid
SGZ	subgranular zone
